# Supplementary figures and images for: The mediation role of sleep on the relationship between drinks behavior and female androgenetic alopecia
Source: PeerJ. 2024 Dec 6;12:e18647. doi: 10.7717/peerj.18647 (PMC11627085; doi:10.7717/peerj.18647)

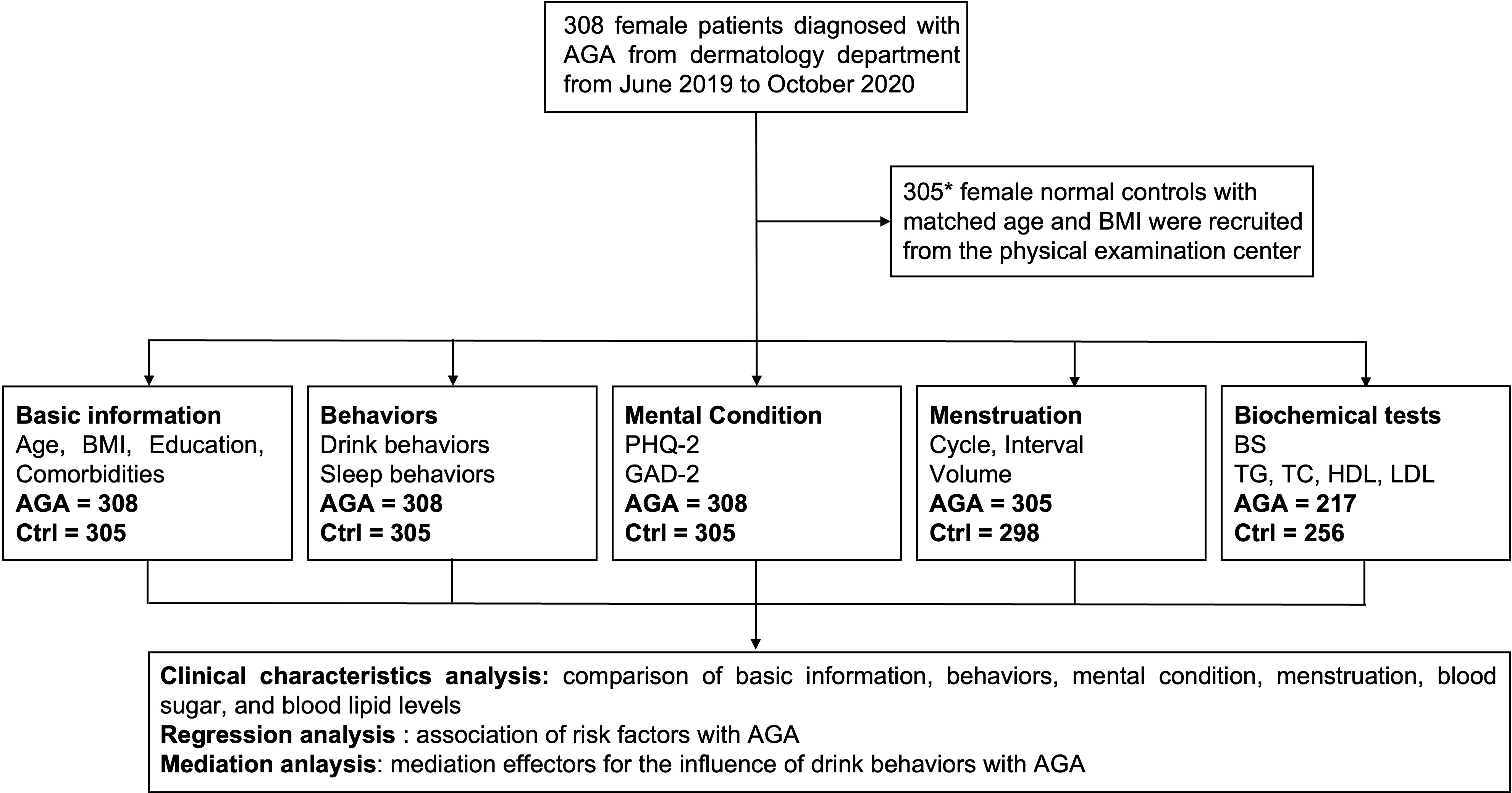

Supplement: Supplemental Information 1 — * calculation of the control group’s sample size was depicted in detail in the 2.1. Study subjects and survey. AGA, androgenetic alopecia; Ctrl, control group; PHQ-2, patient health questionnaire-2, a questionnaire screening for depression; GAD-2, generalized anxiety disorder 2, a questionnaire screening for anxiety; BS, blood sugar; TG, triglyceride; TC, total cholesterol; HDL, high-density lipoprotein; LDL, low-density lipoprotein. [file peerj-12-18647-s001.jpg]
