# Supplementary material for: The mediation role of sleep on the relationship between drinks behavior and female androgenetic alopecia
Source: PeerJ. 2024 Dec 6;12:e18647. doi: 10.7717/peerj.18647 (PMC11627085; doi:10.7717/peerj.18647)
Supplement: Supplemental Information 2 [file peerj-12-18647-s002.docx]

**Supplementary Table 1. Drink behavior in participants**

|  | |  | Ctrl (N=305) | AGA(N=308) | *p* |
| --- | --- | --- | --- | --- | --- |
| Carbonated soda times/wk. | | |  |  |  |
|  | | Hardly | 218, 71.5% | 200, 64.9% | 0.039 |
|  | | 1-2 | 71, 23.3% | 100, 32.5% |  |
|  | | 3-6 | 14, 4.6% | 7, 2.3% |  |
|  | | ≥7 | 2, 0.7% | 1, 0.3% |  |
| Sweetened tea drinks times/wk. | | |  |  |  |
|  | | Hardly | 196, 64.3% | 147, 47.7% | <0.001 |
|  | | 1-2 | 93, 30.5% | 138, 44.8% |  |
|  | | 3-6 | 15, 4.9% | 23, 7.5% |  |
|  | | ≥7 | 1, 0.3% | 0 |  |
| Fruit-flavored drinks times/wk. | | |  |  |  |
|  | | Hardly | 212, 69.5% | 209, 67.9% | 0.541 |
|  | | 1-2 | 88, 28.9% | 89, 28.9% |  |
|  | | 3-6 | 5, 1.6% | 9, 2.9% |  |
|  | | ≥7 | 0 | 1, 0.3% |  |
| Coffee | times/wk. | |  |  |  |
|  | | Hardly | 237, 77.7% | 235, 75.3% | 0.592 |
|  | | 1-2 | 49, 16.1% | 50, 16.2% |  |
|  | | 3-6 | 17, 5.6% | 25, 8.1% |  |
|  | | ≥7 | 2, 0.7% | 1, 0.3% |  |
| Tea | times/wk. | |  |  |  |
|  | | Hardly | 182, 59.7% | 194, 63.0% | 0.346 |
|  | | 1-2 | 79, 25.9% | 79, 25.6% |  |
|  | | 3-6 | 32, 10.5% | 30, 9.7% |  |
|  | | ≥7 | 12, 3.9% | 5, 1.6% |  |
| Milk | times/wk. | |  |  |  |
|  | | Hardly | 61, 20.0% | 53, 17.2% | 0.371 |
|  | | 1-2 | 113, 37.0% | 134, 43.5% |  |
|  | | 3-6 | 105, 34.4% | 93, 30.2% |  |
|  | | ≥7 | 26, 8.5% | 28, 9.1% |  |
| Daily water  intake except drinks L | | |  |  |  |
|  | | <1 | 95, 31.1% | 110, 35.7% | 0.674 |
|  | | 1-1.5 | 155, 50.8% | 144, 46.8% |  |
|  | | 1.6-2 | 44, 14.4% | 44, 14.3% |  |
|  | | >2 | 11, 3.6% | 10, 3.2% |  |
