# Supplementary material for: The mediation role of sleep on the relationship between drinks behavior and female androgenetic alopecia
Source: PeerJ. 2024 Dec 6;12:e18647. doi: 10.7717/peerj.18647 (PMC11627085; doi:10.7717/peerj.18647)
Supplement: Supplemental Information 3 [file peerj-12-18647-s003.docx]

**Supplementary Table 2. Comparison of blood sugar and blood lipids between F-AGA and control**

|  | Ctrl (N=256) | AGA(N=217) | *p* |
| --- | --- | --- | --- |
| Blood Sugar | 5.02±0.43 | 5.12±0.46 | 0.023 |
| TG | 1.05±0.56 | 0.89±0.41 | <0.001 |
| TC | 4.67±0.97 | 4.57±0.80 | 0.223 |
| HDL | 1.47±0.28 | 1.44±0.24 | 0.222 |
| LDL | 2.85±0.71 | 2.78±0.58 | 0.218 |
