# Supplementary material for: The mediation role of sleep on the relationship between drinks behavior and female androgenetic alopecia
Source: PeerJ. 2024 Dec 6;12:e18647. doi: 10.7717/peerj.18647 (PMC11627085; doi:10.7717/peerj.18647)
Supplement: Supplemental Information 4 [file peerj-12-18647-s004.docx]

**Supplementary Table 3. Comparison of blood sugar and blood lipids in F-AGA of different disease severity**

|  | Mild (Savins I) (N=232) | Moderate/severe (Savins II and above) (N=75) | *p* |
| --- | --- | --- | --- |
| Blood Sugar | 5.07±0.42 | 5.29±0.56 | 0.003 |
| TG | 0.83±0.34 | 1.07±0.56 | 0.006 |
| TC | 4.52±0.74 | 4.71±0.98 | 0.22 |
| HDL | 1.45±0.24 | 1.40±0.23 | 0.176 |
| LDL | 2.74±0.53 | 2.90±0.72 | 0.165 |
